# Ultrasound-guided injection into the lateral crico-arytenoid muscle: a pilot study

**DOI:** 10.1007/s00405-023-07843-y

**Published:** 2023-02-11

**Authors:** Berit Schneider-Stickler, Guan-Yuh Ho, Bernhard Moriggl

**Affiliations:** 1 Division of Phoniatrics, Department of Otorhinolaryngology, Medical University of Vienna, Waehringer Guertel 18-20, 1090 Vienna, Austria; 2 Institute of Clinical and Functional Anatomy, Medical University of Innsbruck, Innsbruck, Austria

**Keywords:** Botulinum neurotoxin, Injection of botulinum toxin, Spasmodic dysphonia, Voice tremor, Contact granuloma, Laryngeal synkinesis

## Abstract

**Objectives:**

The anterior, percutaneous Botulinum neurotoxin (BoNT) injection in the lateral cricoarytenoid muscle (LCA) guided by laryngeal electromyography (LEMG) is considered the golden standard treatment for several neurolaryngological disorders. The study presented in this article aims to assess the effectiveness of an alternative approach by which the injection is performed laterally under ultrasound monitoring.

**Study design:**

Anatomical dissection study in human cadavers.

**Settings:**

Academic health care center.

**Methods:**

Ultrasound-guided bilateral dye (0.1 mL of dye solution containing cold-curing polymers, latex, acrylates, acrylic esters, alcohol, and green color) injection in the LCA was performed by means of 24G needles and 1 mL syringes using the lateral approach. The dye location and distribution were assessed by anatomic dissection, performed immediately after the injection.

**Results:**

In 9/10 specimens, the dye was exclusively detectable in the LCA. In 1/10 case (left side), the dye could not be delivered in the LCA because of unintended penetration of the thyroid cartilage by the needle during injection. Anatomic dissection confirmed that the dye spread neither into the thyroarytenoid (TA) nor the cricothyroid muscle (CT).

**Conclusions:**

The anatomic dissection following lateral dye injection in the LCA under ultrasound guide confirmed the precision of this approach in delivery a substance exclusively in a pre-determined target. This feature makes this method an interesting addition or alternative to the standard LEMG-guided BoNT injection at least when the LCA is its target.

**Level of evidence:**

III.

## Introduction

**Fig. 1:**
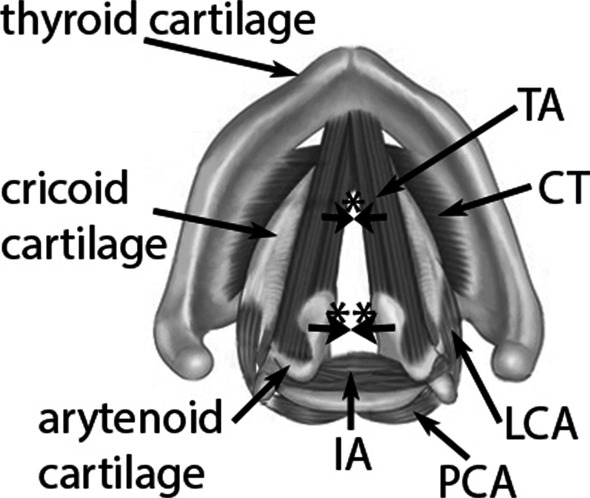
Axial sketch of the laryngeal framework and muscles. *TA* thyro-arytenoid muscle, *CT* crico-thyroid muscle, *LCA* lateral crico-arytenoid muscle, *PCA* posterior crico-arytenoid muscle, *IA* interarytenoid muscle. *Adductorial effect of the TA; **Adductorial effect of the LCA

The lateral cricoarytenoid muscle (LCA) is one of the adductor muscles behind phonation. Together with the interarytenoid muscle (IA), the LCA contraction controls the closure of the glottal cartilaginous portion between the vocal processes and the posterior portion of the larynx, whereas the thyroarytenoid muscle (TA) plays an important role in the adduction of the anterior portion of the respective vocal fold (Fig. 1).

In 2014, Yin and Zhang showed that the LCA activation is behind the vocal fold medial rotation through its shortening and stiffening, toward the glottal midline, which counteract the rocking motion of the arytenoid cartilages [1].

In 2015, Chhetri and Neubauer published new results about the respective role of TA and LCA in phonation [2]. The authors concluded that LCA activation controls the posterior glottal and the TA activation the membranous glottal closure, suggesting that a complete glottal closure requires the simultaneous bilateral activation of both muscles.

Pathologically increased LCA activity compared to TA activity usually results in forceful cartilaginous contact near the tip of the vocal processes, and can be diagnosed electromyographically in several neurolaryngological disorders, such as voice tremor (VT), adductor type of spasmodic dysphonia (AdSD) and some types of synkinetic reinnervation after unilateral/bilateral recurrent laryngeal nerve (RLN) paresis. It can also lead to persistent vocal process granulomas [3].

Currently, transcutaneous botulinum neurotoxin (BoNT) injection in either or both the TA and the LCA is considered the golden standard treatment to reduce the symptoms caused by overactive or synkinetically reinnervated laryngeal muscles. Patients suffering from such disorders generally benefit from the muscle chemodenervation achieved by targeted BoNT injection, by inhibiting the release of acetylcholine at the neuromuscular junction and in cholinergic sympathetic and parasympathetic neurons [4–10].

For decades, laryngeal electromyography (LEMG) has been the worldwide preferred method to ensure the targeted administration of BoNT in patients suffering from neurolaryngological disorders [11–13], as confirmed by Shoffel-Havakuk et al. in 2019. In this work, the authors evaluated the results of a national survey on BoNT injection for AdSD in the USA [14]. The survey revealed that 88% of the laryngologists performed BoNT injections under LEMG guidance via cricothyroid membrane, 9% using anatomical landmarks, and 3% using endoscopic guidance.

The transcricothyroid membrane approach to the intrinsic laryngeal muscles was described the first time in 1969 by Hirano et Ohala [15]. The authors illustrated how to reach the muscles by needle insertion (via hooked-wire electrodes) through the cricothyroid space, penetrating the cricothyroid membrane anterior and reaching the inferior tubercle of the thyroid cartilage. The needle should be pushed first posteriorly, and then slightly laterally and upward, until it reaches the LCA. This approach is ideal when the target of the BoNT is the TA, which is much more frontal than the LCA. Still, the latter muscle is often the chosen target for the treatment of neurolaryngological diseases that affect the adduction of the vocal folds. In the case of LCA-targeted BoNT injection, a frontal percutaneous approach is little effective, since the needle cannot easily reach this muscle, which is more lateral and posterior compared to the TA, for which the technique was initially developed.

To date, very few applications of ultrasound in neurolaryngology are reported worldwide. For instance, to our best knowledge only 2 case-series have been published on ultrasound-assisted transcutaneous injection in the vocal folds for the treatment of unilateral vocal cord by augmentation [16, 17]. In general, in spite of its excellent safety profile, this ultrasound use is limited to very obese patients or to patients with calcified cartilages, for whom other approaches are expected to be far less effective. Interestingly, ultrasound imaging (B-mode) is widely used to examine laryngeal structures and their functionality [18], as well as to locate nerves and assess the effectiveness of different nerve-blockers, as it is the case of ultrasound-guided bilateral superior laryngeal nerve block to facilitate awake fiberoptic intubation [19], cervical plexus blocks for head and neck operations [20], and blocking the internal branch of the superior laryngeal nerve to anesthetize the root of tongue, the epiglottis, and the laryngeal mucosa above the glottis fissure [21]. Ultrasound is also extensively used in cricothyrotomy [22, 23] and in laryngeal diseases unrelated to recurrent laryngeal nerve injuries/dysfunctions? [24]. Since the 1970ies, ultrasound has gained more and more clinical relevance in the management of swallowing disorders, since it is particularly useful for the detailed evaluation of structures and dimensions of the tongue and of the hyoid bone, as well as for the assessment and quantification of laryngeal muscle activity. [25, 26]

This article describes the assessment of an alternative approach to the frontal percutaneous LEMG-guided BoNT injection in the LCA that consists in a lateral percutaneous injection under ultrasound monitoring. In particular, our work focuses on the target precision and specificity of the method.

## Methods

The study was performed at the Institute of Clinical and Functional Anatomy, Medical University of Innsbruck (MUI) in cooperation with the Division of Phoniatrics and Logopedics of the Medical University of Vienna.

Body donors and dye solution.

The experiment described in this article was conducted in five donated cadavers (3 female, 2 male, age range 74–88 years; BMI range 19–28). Each and all donors signed an official informed consent for cadaver donation for research purposes. According to XXX law, all cadavers were in legal custody of the Institute of Clinical and Functional Anatomy, MUI [27, 28].

The cadavers were embalmed with an Ethanol/Glycerol/Phenol solution. Using this embalming method, flexibility of donated corpses comes near to that of the living and allows reliable ultrasound visualization of anatomical structures [29].

0.1 mL of dye solution containing cold-curing polymers, latex, acrylates, acrylic esters, alcohol, and green color was injected in both LCAs by means of 24G needles and 1 mL syringes using the lateral approach under ultrasound monitoring.

### Ultrasound guided dye injection into the LCA

Ultrasound monitoring was performed by means of the Venue2 (company GE) with a linear 13 MHz probe. The transducer was applied with the pre-set “musculoskeletal”, and “superficial nerve” was chosen for imaging.

**Fig. 2:**
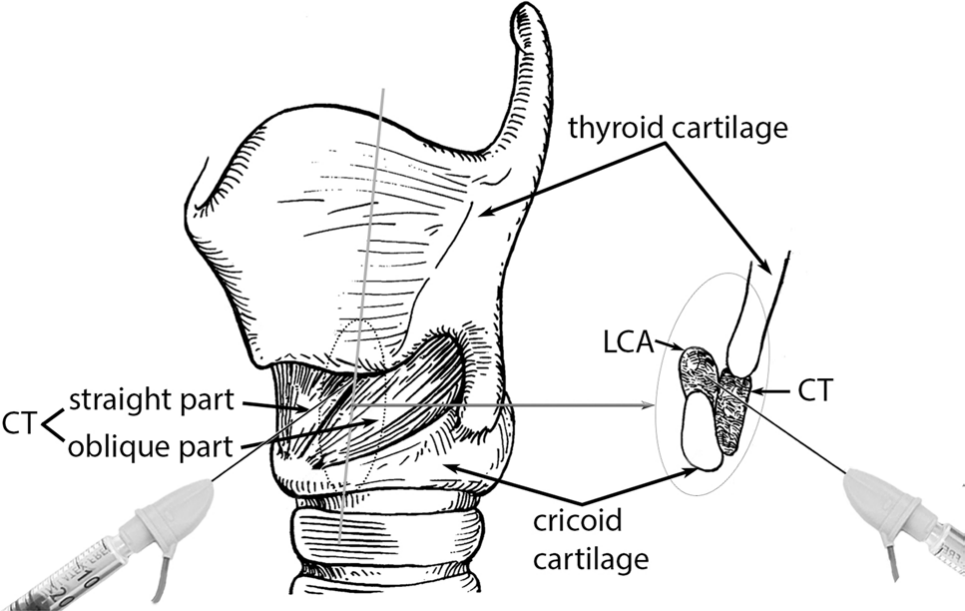
Anatomical situation of needle insertion into the LCA e (modified after Hirano et Ohala, 1969) [15]. *LCA* lateral crico-arytenoid muscle, *CT* crico-thyroid muscle

The operator decided for reaching the LCA through the cricothyroid muscle (CT) under ultrasound monitoring based on the anatomy of the cadavers (Fig. 2). The ultrasound was used to detect the lowest portion of the LCA caudally to the inferior border of the thyroid cartilage lamina through the CT.

**Fig. 3:**
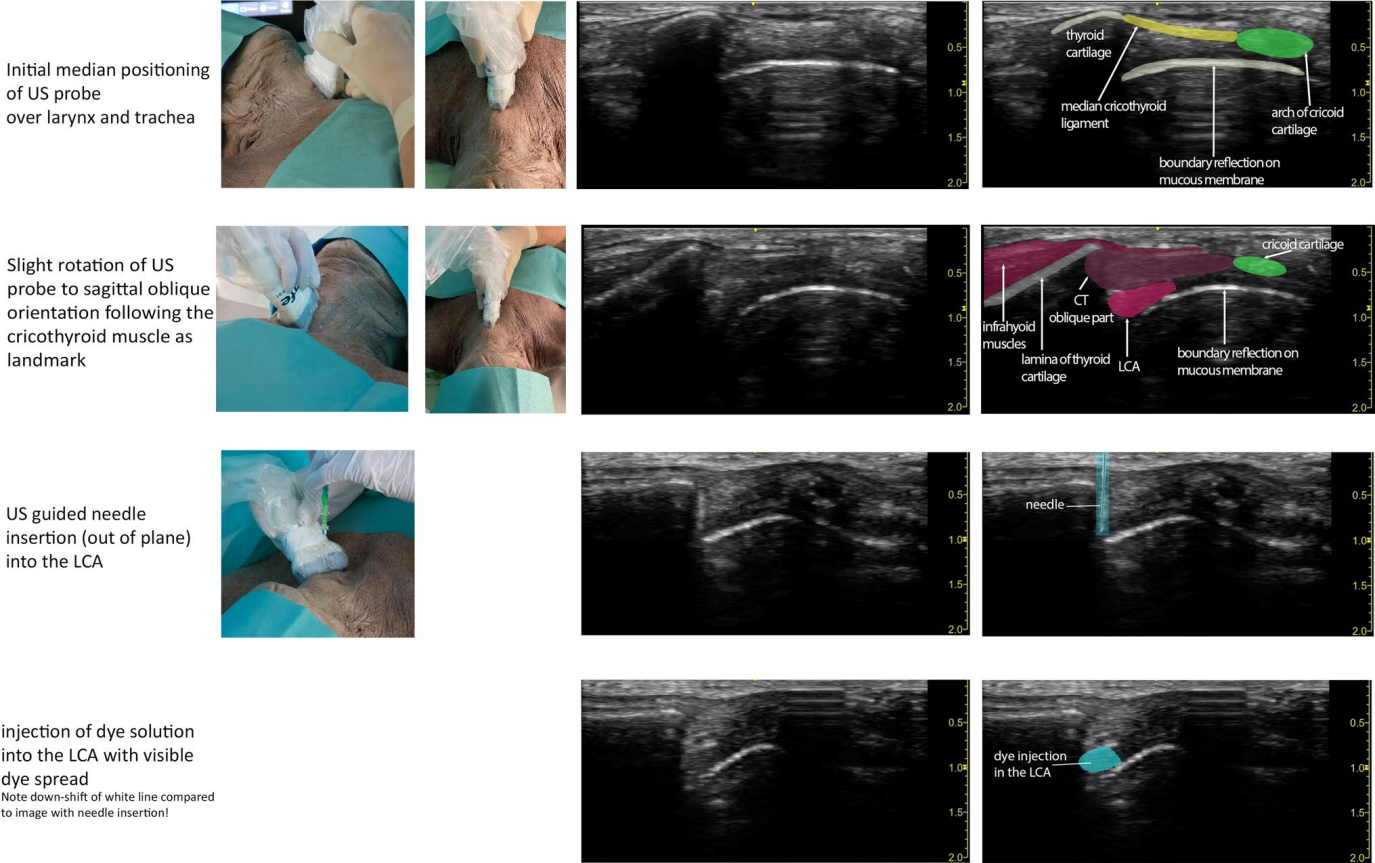
Overview of probe positioning and needle insertion with corresponding ultrasound image

As depicted in Fig. 3, the initial position of the ultrasound probe was based on typical anatomic landmarks, such as the thyroid cartilage, the cricothyroid membrane, and the cricoid cartilage, identified by palpation and visual observations on the midline between the larynx and the trachea. At this point, the probe was slightly rotated to a paramedian oblique orientation with permanent visualization of the CT as muscular landmark. After identification of the straight and the oblique portions of the CT, the lowest portion of the LCA was detected. Under ultrasound guidance, the needle was inserted out of plane into the LCA. Real-time ultrasound was used to follow the dye spreading into the target muscle upon injection.

### Dissection

**Fig. 4:**
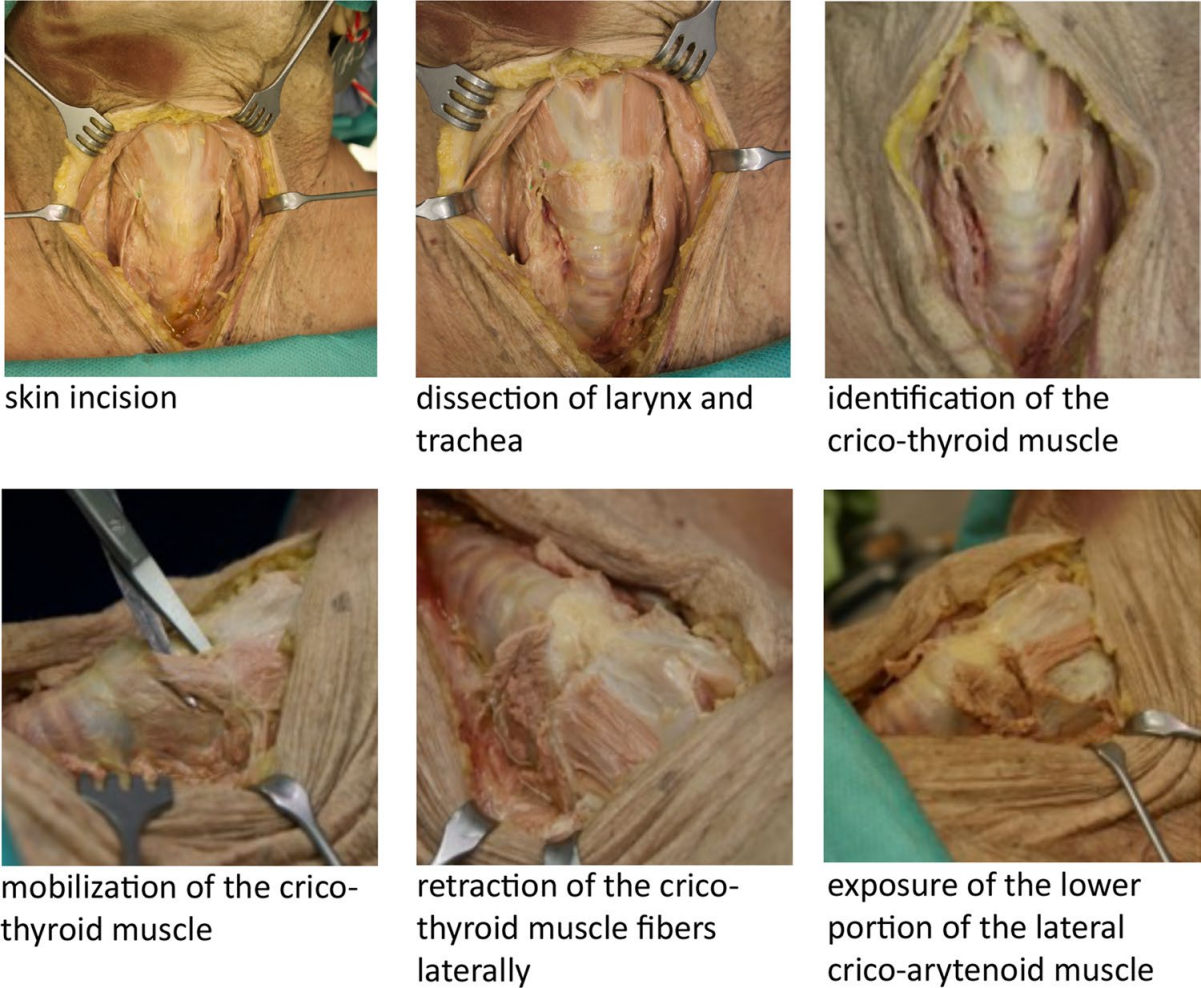
Steps of cadaver dissection. *No dye injection of the left LCA because of injection failure due to needle clogging after unwanted cartilage penetration (donor #2)

Immediately after injection, each cadaver was dissected as depicted in Fig. 4. A median incision was performed from the hyoid bone to the sternum. The incision cut through all the anatomic layers down to the thyroid and cricoid cartilages as well as the tracheal rings. The infrahyoid muscles were laid free and then lateralized to expose both CTs. After mobilization of the CT fibers from the lower thyroid rim, the muscle bellies were pulled downward. Finally, the lowest portion of the LCA was exposed to evaluate the path and the area of the dye spreading.

During dissection special emphasis was given to any dye spread along the needle insertion canal. Any possible dye spreading into prelaryngeal tissue layers or into the CT was assessed and documented before the LCA was exposed. Furthermore, the possible dye spreading into the TA was assessed after identification of the dye deposits in the LCA.

## Results

During dissection, we observed that a peculiar pyramidal lobe and levator muscle of the thyroid gland in the donated cadaver #4. #5 showed a massively enlarged thyroid.

**Fig. 5:**
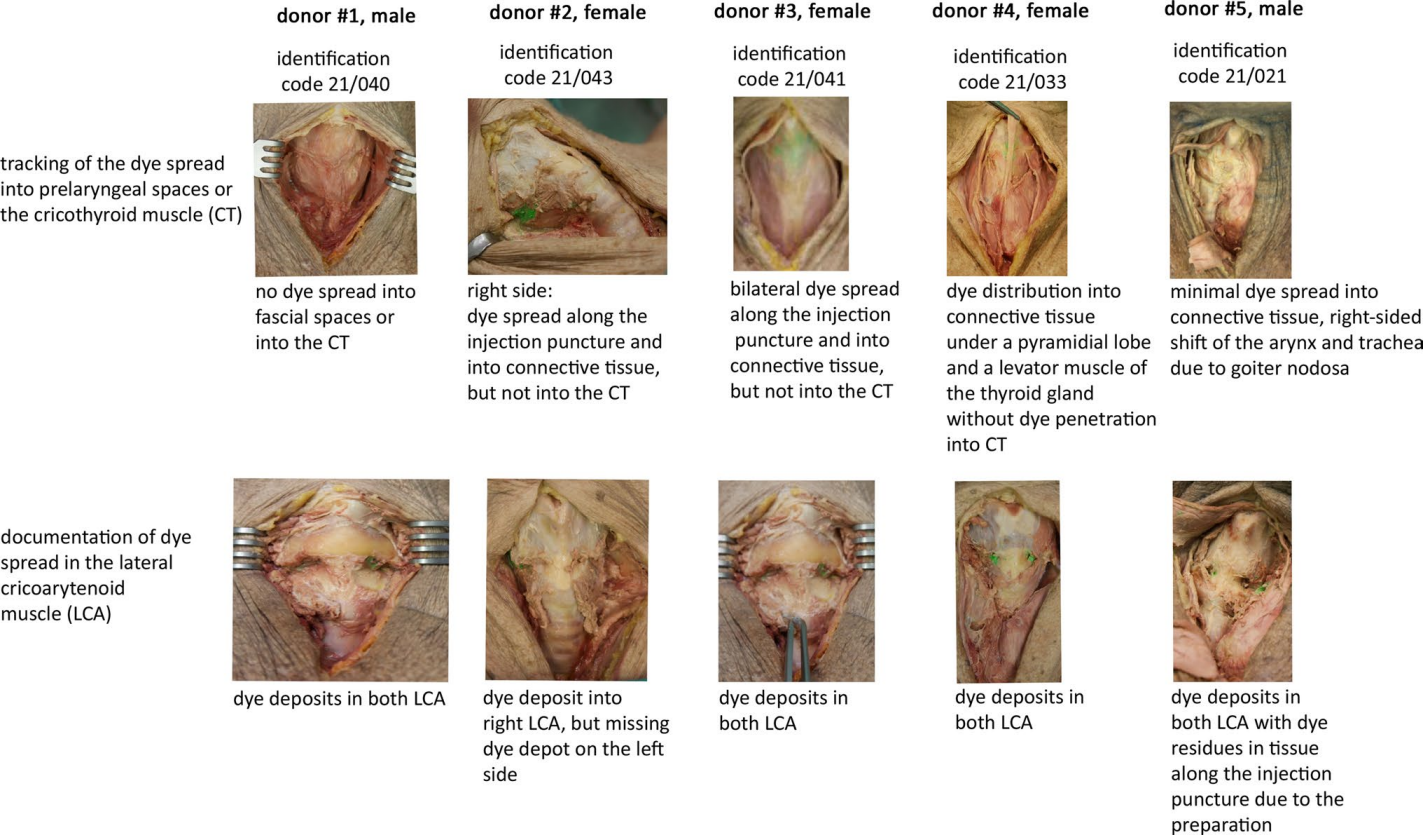
Results of dye deposits after anatomical dissection

Figure 5 depicts the dissection results concerning dye spreading for each of the donated cadavers. In short, careful bilateral dissection failed to detect any dye spreading into the CT before exposing the LCA. The dissection revealed minimal spreading within connective tissue along the needle path.

In 9/10 dissected LCAs, the dye was found exclusively within the LCA. In 1/10 case (#2), the dye could not be delivered in the LCA because of unintended penetration of the thyroid cartilage by the needle during injection. Anatomic dissection confirmed that the dye spread neither into TA nor CT muscles.

## Discussion

Our cadaver study showed for the first time that ultrasound-guided sideway injection of a dying substance in the LCA is effective and precise, suggesting that such method could become a valid alternative to only LEMG controlled transcutaneous BoTN injection in this muscle. In four out of five cadavers, the dye was successfully deposited into the substance of the LCA on both sides. No spreading occurred into the TA and CT, respectively. Only on one side of the remaining cadaver, the injection failed due to needle clogging after unintentional cartilage penetration.

The results showed that bilateral US monitored injection can be successfully conducted without requiring extra-training by the operator. This technique is extremely precise, as confirmed by our observation that the dye did not reach untargeted muscles close to the LCA, such as the CT or the TA. Still the main problem is broadening the use of the ultrasound beyond the common imaging for diagnostic purposes. In the ENT field, in particular, its use is assisting therapeutic approaches is limited to special cases for which no valid alternatives exist, such as the treatment of patients suffering from obesity or calcified thyroid cartilage. This issue is strictly linked to the additional challenges that the application of sonography poses to its application in the ENT field [20].

The results of this study support the clinical need for networking between the expertise in ultrasound application in ENT and the LEMG application established in neurolaryngology. It shall be noted that the use of ultrasound for the monitoring of the LCA injection of BoTN does not require special probes or settings. This, together with its effectivity and precision should enable an increased used in the field of neurolaryngology at least. Clinical studies concerning the use of the ultrasound in combination with LEMG for guiding BoTN injection would provide a more detailed picture of the technique and allow effective exchanges among experts that should support the development of a standardized protocol.

## Conclusion

Our results confirmed the effectiveness and precision of ultrasound-guided BoNT injection into the LCA. Thus, it should be considered as a useful alternative or addition to LEMG-monitoring, especially when the target of the injection are laryngeal muscles that are not easily reachable by means of a frontal, percutaneous approach.

## Data Availability

The data is on file with the corresponding author and is available here.
